# Female Doctors

**Published:** 1881-09

**Authors:** 


### Female Doctors.

The London *Lantern* is by no means a rival of the *Lancet* as an authority on strictly medical questions. This fact, however, does not prevent it from having views. Its opinion on the question of woman in medicine is based on a view of the question not much dwelt on by more eminent medical and medico-ethical authorities. We present it as a piece of pleasant reading:

"This is a subject we approach with great trepidation. We admit that; but the programme we announced in the first issue of the *Lantern* must be fulfilled. We, therefore, characterize the steady increase in the number of female doctors as an alarming sign of the times. We set our face against female doctors, and shall continue to do so until age compels us to resign our functions. If the female doctors were limited to one or two, we should not feel so much exacerbated, but as they are multiplying in all parts of the kingdom, duty impels us to come forward and protest against the innovation, and in so doing we will give a few reasons why female doctoring is not likely to prove a paying business.

"In the first place, if female doctors are to seriously doctor anybody, it must be women, and we strongly suspect that, as a rule, women prefer male doctors. On the other hand, a man, if there was nothing the matter with him, might call in a female doctor; but if he was sick as a horse—and when a man is sick, he *is* sick as a horse—the last thing he would have about would be a female doctor. And why? Because, when a man wants a female fumbling around him, he wants to feel well. He doesn't want to be bilious, or feverish, with his mouth like a furnace and his eyes bloodshot, when a female is looking him over and taking an account of stock. Of course, the coming female doctors will be all young and good looking, and if one of them came into a sick-room where a man was in bed, and he had chills, and was as cold as a frog, and she should sit up close to the side of the bed and take hold of his hand, his pulse would run up to a hundred and fifty, and she would prescribe for a fever when he had chilblains. Then if he died she could be arrested for malpractice. A man who has been ill and had male doctors, knows just how he would feel to have a female doctor come tripping in and throw her fur-lined cloak over a chair, take off her hat and gloves and throw them on a lounge, and come up to the bed with a pair of marine-blue eyes, with a twinkle in the corner, and look him in the face and ask him to exhibit his tongue. Suppose he knew his tongue looked like a Turkish towel, do you suppose he would want to run out five or six inches of the lower end of it and let that female doctor examine it? Not much. He would put that tongue up into his cheek and wouldn't let her see it for half a guinea admission.

"Then again, we are under the impression that male doctors sometimes put their hands under the bed clothes and feel of a man's feet, to see if they are cold. If a female doctor should do that it would give a man the cramps in the legs. It would make a man worse—much worse, and he would want to get up and kick himself for employing a female doctor. Or, suppose a man had heart disease, and a female doctor should want to listen to the beating of his heart. She would lay her left ear on his left breast, so her eyes and rosebud mouth would be looking right in his face, and her wavy hair would be scattered all around there, getting tangled in the buttons of his night-shirt. Don't you suppose his heart would get in about twenty extra beats to the minute? Rather! And she would smile—we bet a fiver she would smile—and show her pearly teeth, and her ripe lips would be working as though she were counting the beats, and he would think she were trying to whisper to him, and—. Well, what would *he* be doing all this time? If he was not dead yet, which would be a wonder, his left hand would brush the hair away from her temple, and just stay there to keep the hair away, and his right hand would get sort of nervous and move around to the back of her head, and when she had counted the heart beats a few minutes and was raising her head, he would draw the head up to him and kiss her once for luck, if he was as bilious as a boiled owl, and have her charge it in the bill. And then a reaction would set in, and she would have to fan him, and rub his head till he got over being nervous, and then make out her prescription after he got asleep. No; a man's symptoms change completely when a female doctor is practicing on him, and she would kill him dead.

"The *Lantern* is a woman's rights paper, and believes in allowing women to do anything that they can do as well as men, and is in favor of paying them as well as men are paid for the same work, taking all things into consideration, but it is opposed to their trifling with human life by trying to doctor a total stranger.

"That is why we are sternly opposed to the innovation, and, as we said before, we will set our face against female doctors until we are old and toothless."—*Michigan Medical News.*

